# Peripheral biomarkers to assess risk, severity, and prognosis of immune checkpoint inhibitor-associated myocarditis: a retrospective clinical study

**DOI:** 10.3389/fcvm.2024.1465743

**Published:** 2024-10-24

**Authors:** Zhengkun Guan, Tiezhu Yao, Guang Liu, Jing Liu, Ling Guo, Zhenli Li, Jingtao Ma

**Affiliations:** Department of Cardiology, The Fourth Hospital of Hebei Medical University, Shijiazhuang, China

**Keywords:** immune-checkpoint inhibitor, cardiotoxicity, myocarditis, biomarkers, risk, prognosis

## Abstract

**Background:**

Immune checkpoint inhibitor-associated myocarditis (ICI myocarditis) is an infrequent but potentially fatal immune-related adverse event. This study aimed to identify valuable indicators for risk prediction and evaluation of disease severity and outcomes.

**Methods:**

A total of 79 patients with severe or mild ICI myocarditis and 158 controls without post-ICI immune-related adverse events were enrolled in this retrospective study. The clinical application value of a series of simple biomarkers were tested.

**Results:**

Higher levels of the systemic immune-inflammation index (SII), neutrophil-to-eosinophil ratio (NER), aspartate transferase-to-albumin ratio (AAR), and lactic dehydrogenase-to-albumin ratio (LAR) at myocarditis onset were associated with severe disease conditions. In the receiver operating characteristic analysis, biomarkers areas under the curve (AUC) close to or greater than 0.8 were LAR (AUC: 0.810) and AAR (AUC: 0.806). Patients with higher SII, AAR, and LAR also exhibited poorer overall survival. The SII, NER, AAR, and LAR before the last ICI treatment increased relative to baseline in patients with ICI myocarditis, whereas no significant changes in the tested biomarkers were observed in the control group. For SII, AAR, and LAR, high ratios of the biomarker levels before the last ICI to baseline was associated with the incidence of myocarditis.

**Conclusions:**

Surveillance of these economical biomarkers during ICI therapy might contribute to the risk prediction of ICI myocarditis, as well as the assessment of disease severity and prognosis.

## 1 Introduction

The advent of immune checkpoint inhibitors (ICIs) has triggered a paradigm shift in oncology. Despite their therapeutic efficacy, the administration of ICIs may give rise to immune-related adverse events (irAEs) that can impact multiple organs, including those of the cardiovascular system (1, 2). Immune checkpoint inhibitor-associated myocarditis (ICI myocarditis) is a rare irAE, with a recently reported incidence of 0.3%–1.7% (3, 4). However, ICI myocarditis exhibits a significantly higher mortality rate of 39%–50% in comparison to other irAEs (5, 6). Owing to the growing use of ICIs, awareness regarding the clinical presentation, suspicion, diagnosis, and management of ICI myocarditis must be raised.

The manifestations of ICI myocarditis vary from asymptomatic states to life-threatening outbreaks (7), including fatigue, palpitations, chest pain, orthopnea, edema, syncope, cardiogenic shock, and so forth (8–10). The development of myocarditis not only leads to the discontinuation of ICI treatment but also endangers patients’ lives, especially in severe cases (9, 11). Therefore, prompt assessment of the severity and prognosis of ICI myocarditis is crucial. The use of cardiovascular magnetic resonance imaging (CMR) can aid in assessing the condition and prognosis of patients with myocarditis (12, 13); however, the complexity of the procedure limits its widespread use. Although severe arrhythmias often indicate a grave condition and an unfavorable prognosis, certain electrocardiogram (ECG) manifestations lack specificity (14). Despite the association of some echocardiography parameters with the occurrence of adverse events (15), the role of echocardiography appears to be limited (16). The presence of elevated cardiac markers can indicate myocardial injury and impaired cardiac function, which have been associated with severe myocarditis and worse outcomes. Nevertheless, not all patients manifest elevated levels of cardiac markers (17–19). And these markers are sometimes also affected by other factors, such as acute coronary syndrome, heart failure, and severe renal dysfunction (10). To date, other indicators that have been proposed to guide disease assessment and predict mortality are still limited.

Another critical clinical task is to identify patients susceptible to ICI myocarditis. Currently, only a few possible risk factors have been reported. Compared with monotherapy, the incidence of myocarditis reported for combination therapy with ICIs is higher (5, 16). Mounting evidence suggests that pre-existing cardiovascular diseases represent potential risk factors for cardiovascular immune-related adverse events, especially ICI myocarditis (20). The immune response mediated by T cells is a key factor in the occurrence of myocarditis, but the exact pathogenesis is still unclear (21). So far, there are no specific and effective biomarkers that can predict the development of ICI myocarditis, and only a few studies have reported potential indicators (22, 23).

Therefore, more indicators need to be explored to guide the risk prediction and evaluation of disease severity and prognosis of ICI myocarditis. Some simple markers, including systemic immune-inflammation index (SII), neutrophil-to-eosinophil ratio (NER), aspartate transferase-to-albumin ratio (AAR), and lactic dehydrogenase-to-albumin ratio (LAR), have been reported in other patient cohorts treated with ICIs (24–26). These indicators have been reported to be related to the occurrence, severity or prognosis of cardiotoxicity or other irAEs. But their relationship with ICI myocarditis has not been fully elucidated. Due to the low incidence rate, studies based on clinical data are still lacking. In this study, we investigated the potential clinical utility of these biomarkers in patients with ICI myocarditis.

## 2 Methods

### 2.1 Study population

In this single-center retrospective study, 79 patients diagnosed with ICI myocarditis at the Fourth Hospital of Hebei Medical University between November 2019 and December 2023 were identified among the 6,778 patients treated with ICIs. ICI myocarditis was diagnosed with reference to the 2021 ICOS Consensus Criteria (27): (1) Pathological diagnosis confirmed by endomyocardial biopsy (EMB). (2) Clinical symptoms accompanied by elevated cardiac troponin levels, as well as CMR or echocardiography findings. (3) Ventricular arrhythmia and/or high-grade conduction system disease with elevated troponin levels. (4) Three of the minor criteria including clinical symptoms, new ECG changes, elevated cardiac troponin, echocardiography findings, immune mediated myositis and suggestive cardiac MRI. (5) Newly elevated troponin, reduced LVEF and/or suggestive CMR, after exclusion of other potential etiologies. The severity of ICI myocarditis was also classified according to the2021 ICOS Consensus Criteria (27), with grades 2 (moderate) defined as mild myocarditis (*n* = 35) and grades 3–4 (including severe and life-threatening) defined as severe myocarditis (*n* = 44). Another 158 patients treated with ICI without any irAEs during the same period were randomly selected as the control group. To further mitigate the impact of confounding factors on the biomarkers, we conducted propensity score matching between the myocarditis group and the control group, and a total of 75 pairs of matched patients were generated. The diagnosis of myocarditis and other irAEs were made by two specialists based on the clinical evidence. This study was approved by the Ethics Committee of the Fourth Hospital of Hebei Medical University.

### 2.2 Study protocol and data collection

Clinical characteristics of the patients were collected. Peripheral blood data, including absolute neutrophil count (ANC), absolute eosinophil count (AEC), absolute lymphocyte count (ALC), platelet count (PLT), aspartate aminotransferase (AST), lactate dehydrogenase (LDH), and albumin (ALB), were utilized for the calculation of six biomarkers (24–26): SII = ANC × PLT/ALC, NER = ANC/AEC, AAR = AST/ALB, and LAR = LDH/ALB. Parameters were collected from patients with ICI myocarditis at baseline, before the last ICI, and at the onset of myocarditis as well as from patients in the control group at baseline and before the last ICI. The primary endpoint of follow-up was all-cause mortality. The cutoff date for follow-up was March 10, 2024.

### 2.3 Statistical analyses

Continuous variables were compared using the *t*-test or Mann–Whitney *U*-test. Comparisons of categorical variables were performed using the chi-square test. The correlation between the biomarkers and the occurrence and severity of myocarditis was explored using logistic regression. To test the clinical application values of these variables, receiver operating characteristic (ROC) curves were plotted, and the area under the curve (AUC) was calculated. DeLong's test was used to compare the ROC curves for different variables (28). The Kaplan–Meier method was used to determine the overall survival (OS) of patients in each group, while the log-rank test was used to evaluate the differences in patient survival time. The relationship between the biomarkers and OS was investigated using the COX proportional hazards regression model. We conducted propensity score matching based on factors including age, gender, tumor type, coronary heart disease, combined chemotherapy, targeted therapy, and radiotherapy. Pairs of patients in the ICI myocarditis group and control group were derived using 1:1 nearest matching within PS score of 0.02. Statistical analysis was performed using SPSS 29, with the significance level set at *p* < 0.05.

## 3 Results

### 3.1 Biomarkers associated with severe ICI myocarditis

**Figure 1:**
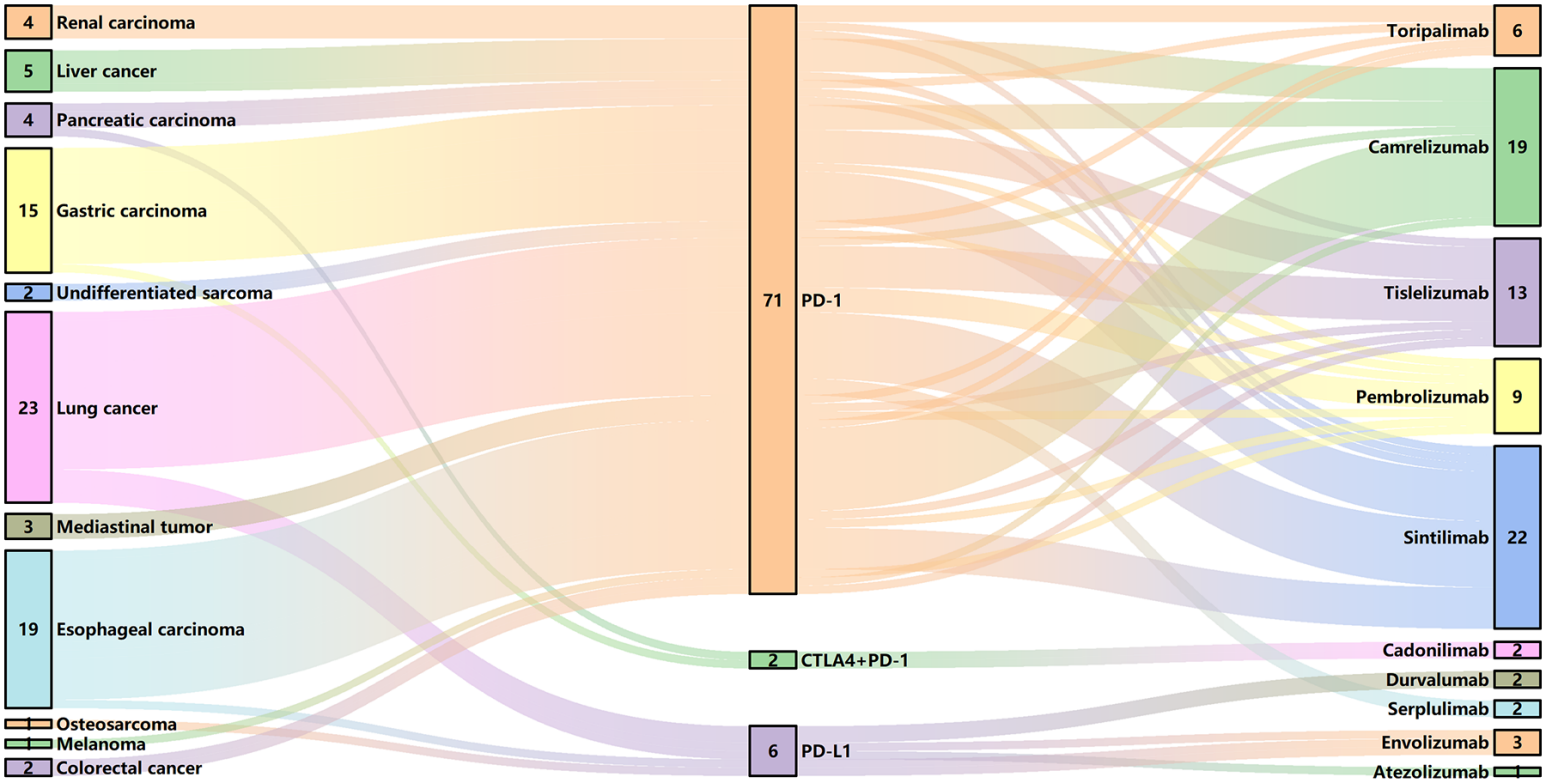
ICI use in patients with myocarditis. CTLA4, cytotoxic T lymphocyte associated antigen 4; PD-1, programmed cell death protein 1; PD-L1, programmed death-ligand 1.

We made a clinical diagnosis of myocarditis in 79 patients based on their clinical symptoms, cardiac MRI, echocardiography, ECG, and changes in cardiac markers, with no evidence of acute coronary syndrome detected through coronary angiography and coronary CTA. However, considering the patient's wishes and physical condition, we did not perform a myocardial biopsy. The most prevalent tumor types in the cohort of 79 patients included lung cancer (23, 29.1%), esophageal carcinoma (19, 24.1%), and gastric cancer (15, 19.0%), with the majority of patients receiving anti-PD-1 therapy (71, 89.9%). The clinical characteristics of patients with mild and severe ICI myocarditis are presented in Table 1 and Figure 1. The median time from the initiation of ICI to the diagnosis of myocarditis was 48 (28, 93) days. Other irAEs were observed in 40 (50.6%) patients, with a higher incidence in the severe group than in the mild group. Myositis is the most frequently concomitant irAEs (Supplementary Figure S1A).

**Table 1:** Clinical characteristics of patients with high-grade and low-grade ICI-associated myocarditis.

Characteristics	All patients *N* = 79	Severe group *n* = 44	Mild group *n* = 35	*p*-value
Age	63.94 (10.09)	64.57 ± 10.711	63.14 ± 9.35	0.536
Male	58 (73.4%)	32 (72.7%)	26 (74.3%)	0.876
BMI, kg/m^2^	23.44 (3.77)	23.74 ± 3.73	23.06 ± 3.83	0.426
Smoking history	34 (43.0%)	18 (40.9%)	16 (45.7%)	0.668
Drinking history	14 (17.7%)	6 (13.6%)	8 (22.9%)	0.286
CHD	11 (13.9%)	8 (18.2%)	3 (8.6%)	0.220
Hypertension	23 (29.1%)	14 (31.8%)	9 (25.7%)	0.553
Diabetes	13 (16.5%)	9 (20.5%)	4 (11.4%)	0.282
Tumor type				0.124
Lung cancer	23 (29.1%)	14 (31.8%)	9 (25.7%)	
Esophageal carcinoma	19 (24.1%)	8 (18.2%)	11 (31.4%)	
Gastric cancer	15 (19.0%)	6 (13.6%)	9 (25.7%)	
Other tumors	22 (27.8%)	16 (36.4%)	6 (17.1%)	
Tumor stage				0.562
≤III	40 (50.6%)	21 (47.7%)	19 (54.3%)	
IV	39 (49.4%)	23 (52.3%)	16 (45.7%)	
Therapy mode				
Combined chemotherapy	64 (81.0)	35 (79.5%)	29 (82.9%)	0.709
Combined targeted-therapy	14 (17.7%)	9 (20.5%)	5 (14.3%)	0.476
Combined radiotherapy	12 (15.2%)	7 (15.9%)	5 (14.3%)	0.842
Type of ICIs				0.433
Anti-PD-1	71 (89.9%)	38 (86.4%)	33 (94.3%)	
Others	8 (10.1%)	6 (13.6%)	2 (5.7%)	
Medication cycles	2 (1,3)	2 (1,2.5)	2 (1,3)	0.226
Time to myocarditis (day)	48 (28,93)	43 (25,73)	53 (31,128)	0.111
Concomitant irAEs	40 (50.6%)	29 (65.9%)	11 (31.4%)	0.002
EF at myocarditis onset (%)	60 (52,64)	58 (51,64)	61 (55,65)	0.099

Abbreviations: ICIs, immune checkpoint inhibitors; BMI, body mass index; CAD, coronary heart disease; PD-1, programmed cell death protein 1; irAEs, immune-related adverse events; EF, ejection fraction.

We compared cardiac markers, including creatine kinase isoenzyme (CK-MB), cardiac troponin-I (cTnI), N-terminal pro-brain natriuretic peptide (NT-proBNP), and the selected biomarkers at the onset of myocarditis in patients with mild and severe myocarditis. Univariate logistic regression analysis showed that these indicators were significantly associated with the occurrence of severe myocarditis (Table 2). After adjusting for sex, age, other irAEs, tumor stage, coronary heart disease, and history of diabetes, the relationship between these indicators and severe myocarditis remained statistically significant (Table 2). To evaluate the discriminatory performance of the different indicators in estimating the severity of ICI myocarditis, we plotted ROC curves for these indicators and calculated the AUC. Among these parameters, LAR (AUC: 0.813, 95% CI: 0.717–0.909) and AAR (AUC: 0.806, 95% CI: 0.712–0.900) exhibited AUC values exceeding 0.8 (Supplementary Figures S2A–C). The AUC of the ROC for AAR and LAR combined was 0.831 (95% CI: 0.744–0.919); however, there was no significant difference compared with that of LAR alone (*p* = 0.559, with Delong's test).

**Table 2:** Logistic regression analysis of biomarkers related to the severity of ICI myocarditis sever ICI myocarditis.

Biomarkers at myocarditis onset	Unadjusted		Adjusted^a^	
	OR (95% CI)	*p*-value	OR (95% CI)	*p*-value
SII	1.001 (1.000, 1.001)	0.002	1.001 (1.000, 1.001)	0.006
NER	1.001 (1.000, 1.003)	0.014	1.002 (1.000, 1.003)	0.016
AAR	1.466 (1.153, 1.864)	0.002	1.464 (1.155, 1.856)	0.002
LAR	1.098 (1.037, 1.161)	0.001	1.095 (1.032, 1.162)	0.003
CK-MB, U/L	1.007 (1.001, 1.013)	0.018	1.007 (1.000, 1.013)	0.032
cTnI, ng/mL	1.286 (1.022, 1.618)	0.032	1.297 (1.016, 1.654)	0.037
NT-pro-BNP, ng/L	1.000 (1.000, 1.000)	0.007	1.000 (1.000, 1.001)	0.002

Abbreviations: ICIs, immune checkpoint inhibitors; irAEs, immune-related adverse events; OR, odds ratio; SII, systemic immune-inflammation index; NER, neutrophil to eosinophil ratio; AAR, aspartate transferase to albumin ratio; LAR, lactic dehydrogenase to albumin ratio; CK-MB, creatine kinase isoenzyme; cTnI, cardiac troponin-I; NT-proBNP, N-terminal pro-brain natriuretic peptide.

^a^ Adjusted for age, sex, other irAEs, tumor stage, history of type 2 diabetes, and coronary artery disease.

### 3.2 Association between biomarkers and prognosis

**Figure 2:**
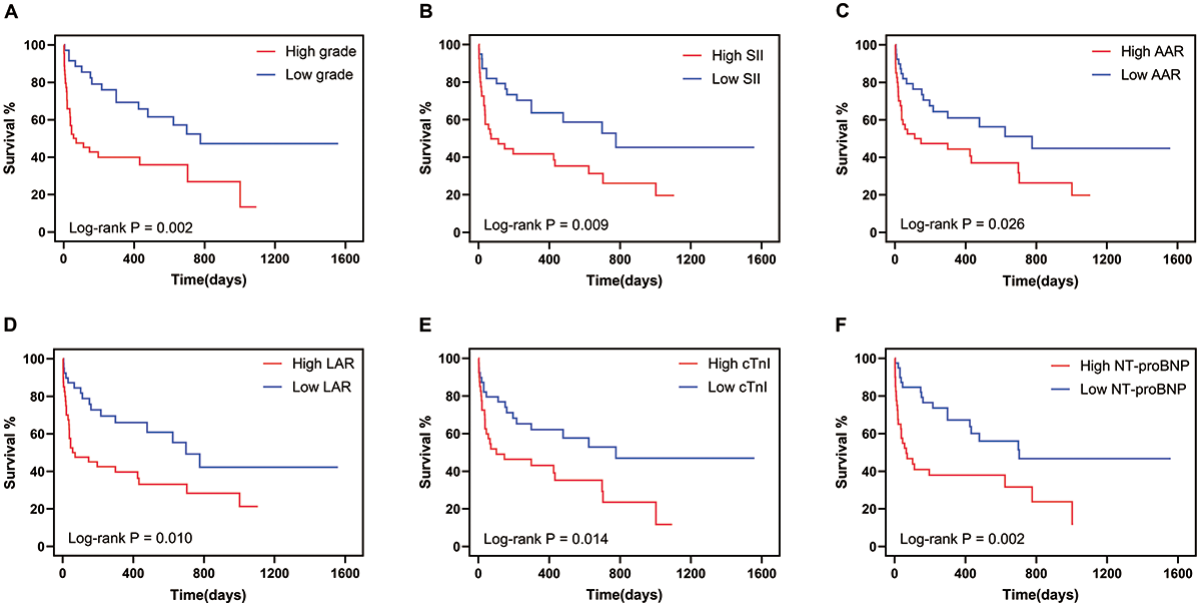
Myocarditis severity and biomarkers associated with all-cause mortality. **(A)** Kaplan–Meier analysis of all-cause mortality in patients with severe myocarditis. **(B–F)** Kaplan–Meier analysis of all-cause mortality in patients above-median and below-median SII **(B)**, AAR **(C)**, LAR **(D)**, cTnI **(E)**, and NT-pro-BNP **(F)** at myocarditis onset. SII, systemic immune-inflammation index; NER, neutrophil to eosinophil ratio; AAR, aspartate transferase to albumin ratio; LAR, lactic dehydrogenase to albumin ratio; CK-MB, creatine kinase isoenzyme; cTnI, cardiac troponin-I; NT-proBNP, N-terminal pro-brain natriuretic peptide.

During a follow-up period of 196 (37,548) days, 44 (55.7%) patients died, with worse OS in the severe group than in the mild group (Figure 2A). BMI was associated with OS (HR 0.917, 95% CI: 0.844–0.995, *p* = 0.038). Patients with advanced tumors had worse OS, but the difference was not statistically significant (299 vs. 776 days, *p* = 0.06, with log-rank test). Univariate Cox analysis showed that the SII, NER, AAR, LAR, cTnI, and pro-BNP levels were all related to OS (Table 3). After adjusting for age, BMI, tumor stage, medication cycles, coronary heart disease, and other irAEs, the association between NER with OS was attenuated, while SII, AAR, LAR, cTnI, and NT-proBNP remained significantly associated with OS (Table 3).

**Table 3:** Cox regression analysis of biomarkers related to the overall survival of ICI myocarditis.

Biomarkers at myocarditis onset	Unadjusted		Adjusted^a^	
	HR (95% CI)	*p*-value	HR (95% CI)	*p*-value
SII	1.000 (1.000, 1.000)	0.001	1.000 (1.000, 1.000)	0.021
NER	1.001 (1.000, 1.002)	0.004	1.001 (1.000, 1.001)	0.065
AAR	1.055 (1.032, 1.077)	<0.001	1.063 (1.036, 1.090)	<0.001
LAR	1.033 (1.020, 1.046)	<0.001	1.035 (1.019, 1.051)	<0.001
CK-MB, U/L	1.000 (0.999, 1.001)	0.716	–	–
cTnI, ng/mL	1.091 (1.046, 1.138)	<0.001	1.104 (1.054, 1.156)	<0.001
NT-pro-BNP, ng/L	1.000 (1.000, 1.000)	<0.001	1.000 (1.000, 1.000)	<0.001

Abbreviations: See Table 2; HR, hazard ratio.

^a^ Adjusted for age, BMI, other irAEs, tumor stage, medication cycles of ICI, and coronary artery disease.

When stratified by the median, patients with higher SII, AAR, LAR, cTnI, and pro-BNP levels exhibited lower OS (Figures 2B–F). We further conducted an analysis of the 44 patients with severe myocarditis. After adjusting for age, BMI, tumor stage, and other irAEs, the association between SII, NER, AAR, LAR, and cTnI with OS remained significantly (Supplementary Table S1). Besides, the control group exhibited superior OS from the initiation of ICI treatment until death compared to the myocarditis group (Supplementary Figure S1B).

### 3.3 Predictors of ICI myocarditis

**Figure 3:**
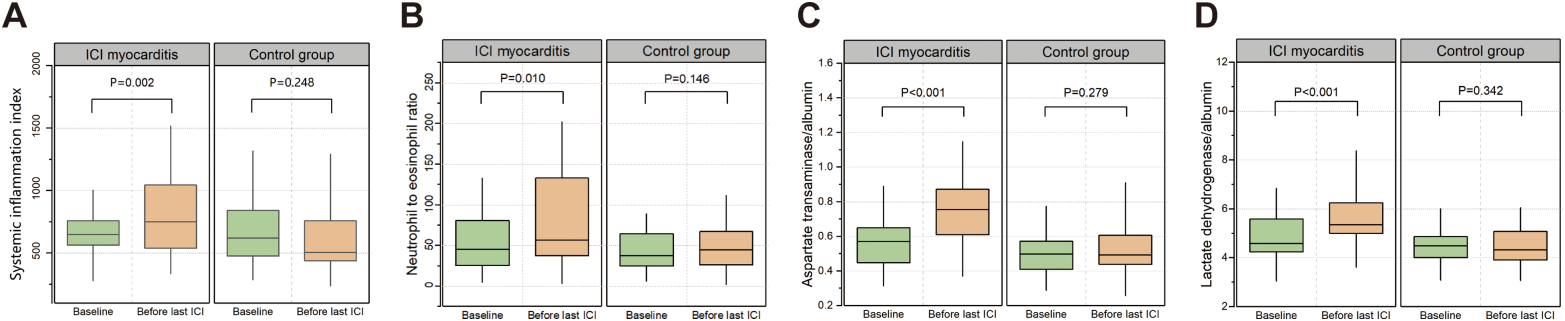
Changes in SII **(A)**, NER **(B)**, AAR **(C)** and LAR **(D)** levels from baseline to the time before last ICI treatment in patients with myocarditis and control participants.

We randomly selected 158 patients who received ICI treatment during the same period without any irAEs as the control group at a ratio of 1:2. Comparison of the clinical characteristics between patients with ICI myocarditis and the control group is presented in Supplementary Table S2. The patients in the control group seemed to be younger, but there were no significant differences in the overall clinical characteristics. In order to better reflect the relationship between the changes of biomarkers and the incidence of myocarditis and eliminate the interference of confounding factors, we conducted propensity score matching based on factors including age, gender, tumor type, coronary heart disease, combined chemotherapy, targeted therapy, and radiotherapy. Pairs of patients in the ICI myocarditis group and control group were derived using 1:1 nearest matching within PS score of 0.02. This strategy resulted in 75 matched pairs in each group. Comparison of the clinical characteristics after propensity score matching is presented in Supplementary Table S3. Compared with the control group, patients in the myocarditis group had a shorter medication cycle and a higher mortality rate. At baseline, there were no significant differences in selected peripheral blood markers between the two groups (Supplementary Table S4). Changes in parameters at baseline and before the last administration of ICIs were compared between patients with myocarditis and control participants. In patients with myocarditis, SII, NER, AAR, and LAR before the last ICI treatment were significantly elevated compared to baseline. Conversely, the indices before the last ICI in the control group showed no significant changes compared with their respective baseline values (Figures 3A–D, Supplementary Table S5). The ratio of these biomarkers before the last ICI treatment to baseline was computed to reflect post-treatment changes. Univariate logistic regression analysis indicated a significant association between myocarditis events and the ratios of SII, AAR and LAR (Table 4). ROC analyses were performed and only AAR (AUC: 0.768, 95% CI: 0.666–0.870) and LAR (AUC: 0.765, 95% CI: 0.678–0.853) had AUCs above 0.7 (Supplementary Figures S2D–E). The combined application of SII, AAR, and LAR yielded an AUC of 0.843 (95% CI: 0.763–0.923), and it was significantly improved from that of AAR alone (*P* = 0.037, with Delong's test). We further conducted an analysis included only patients with myocarditis without other irAEs, this relationship still existed (Table 4).

**Table 4:** Ratio of biomarkers associated with myocarditis.

Biomarkers	Unadjusted		Adjusted^a^	
	OR for ICI myocarditis (95% CI)	*p*-value	OR for ICI myocarditis (95% CI)	*p*-value
SII	2.913 (1.417, 5.989)	0.004	2.340 (1.032, 5.309)	0.042
NER	1.115 (0.946, 1.314)	0.195	1.196 (0.999, 1.432)	0.051
AAR	18.477 (5.033, 67.827)	<0.001	31.749 (6.302, 159.954)	<0.001
LAR	36.024 (5.135, 255.269)	<0.001	30.047 (3.291, 274.356)	0.003

^a^ Analysis after excluding patients with concurrent irAEs. Abbreviations: see Table 2.

## 4 Discussion

In this retrospective analysis, we validated the utility of four simple biomarkers for evaluating the severity and prognosis of ICI myocarditis, as well as their capacity to predict the risk of developing myocarditis. These findings will contribute to the accurate identification of patients with severe disease and poor prognosis and facilitate the identification of potential high-risk groups to enhance cardiovascular surveillance, thereby aiding the early detection and diagnosis of myocarditis.

### 4.1 Assessment of disease severity

In our cohort of patients, those with high-grade myocarditis had worse OS. Therefore, early recognition and treatment of severe myocarditis are crucial. Higher levels of CK-MB, cTnI, and NT-proBNP were associated with higher grades of myocarditis, demonstrating the ability of cardiac markers for risk stratification of patients with myocarditis. ICI myocarditis is characterized by infiltration of immune cells and inflammatory responses (1, 29), the level of inflammatory markers may be related to the severity and prognosis of myocarditis. SII is a novel inflammation marker that encompasses neutrophils, platelets, and lymphocytes, reflecting the body's inflammation and immune status (30, 31). Raziye et al. (32) reported that SII is associated with fulminant myocarditis in young patients. At present, the relationship between SII and ICI myocarditis has not been reported. NER is another inflammation marker that has been reported to be associated with ICI-associated cardiotoxicity (26). Higher levels of neutrophils lead to the production of reactive oxygen species and pro-inflammatory cytokines, which can promote myocardial injury (33). Eosinophils have a cardiac protective effect on the heart after a myocardial infarction (34). An elevated NER may indicate a severe inflammatory response and reduced cardiac protective effects following ICI therapy, which is associated with the severity of myocarditis and a poor prognosis. AST and LDH exhibit widespread distribution within cardiac tissue, and the elevation of these biomarkers serves as a valuable indicator of cardiac injury (35, 36). Albumin reflects nutritional status and plays an important role in inflammation and immune responses. Combining AST and LDH with albumin can simultaneously reflect systemic inflammation, nutritional status, and myocardial injury, thereby helping to assess the severity of ICI myocarditis (37). Our study indicates the relationship between these biomarkers and the severity of ICI myocarditis, the ROC analysis results showed that AAR and LAR had AUCs greater than 0.8, which indicates their promising clinical application value. For patients diagnosed with myocarditis, increases in the AAR and LAR, in addition to cardiac markers, may serve as additional indicators of disease severity and warrant careful attention.

### 4.2 Prognosis of ICI myocarditis

The biomarkers associated with severe myocarditis may also be indicative of an unfavorable prognosis. Giorgi et al. (38) demonstrated a correlation between the SII and OS in a cohort of patients with renal cell carcinoma treated with ICIs. Our study further confirms the broader applicability of the SII by establishing its association with OS in ICI myocarditis. Our study also revealed associations of AAR and LAR with relatively long-term OS compared to the research of Zhuang et al. (24). Neutrophils promote tumor angiogenesis and adhesion of circulating tumor cells by secreting cytokines and chemokines, leading to distant metastasis, tumor cells induce platelet activation through direct contact and secretion of soluble factors, resulting in the adhesion of platelets to tumor cells, thereby enabling evasion of human immune surveillance, Lymphocytes may mediate adaptive immune responses and inhibit the proliferation of malignant cells (39, 40). The level of LDH can reflect the damage caused by tumor cells to tissues and the tumor burden, and is related to the prognosis of cancer patients (41). Consequently, the association between these biomarkers and OS may be influenced by both the severity of myocarditis and the oncological status of the patient. Notably, higher NER is also associated with poor prognosis in patients with severe myocarditis, suggesting that severe inflammation may have a negative impact on prognosis. Besides, cardiac markers can be used to assess prognosis. In our study, cTnI and pro-BNP levels were associated with long-term patient prognosis. The comprehensive utilization of cardiac markers and the SII, AAR, and LAR can contribute to the assessment of long-term OS in patients.

### 4.3 Risk factors

To date, there is no definitive marker to predict the occurrence of ICI myocarditis. Some non-cardiac markers such as alanine aminotransferase, creatine phosphokinase, AST, and LDH increase before the onset of myocarditis, and the development of myocarditis has been associated with changes in these markers (22). Our findings suggest that an elevation in the SII, AAR, and LAR were associated with myocarditis. And the combined application of multiple biomarkers proves to be more valuable than a single indicator in identifying high-risk groups. The elevation of these markers may be linked to the development of other irAEs, indicating a correlation with myocarditis events (22). However, when we included only patients with myocarditis without other irAEs, this relationship still existed. Changes in these biomarkers may reflect underlying immune-inflammatory damage to the heart or other tissues and may be associated with the development of myocarditis, further research is still needed.

### 4.4 Limitations

This study had some limitations. First, this was a single-center retrospective study, which inevitably has a selection bias. Second, no patient in our cohort underwent a subendocardial myocardial biopsy, the gold standard for diagnosis despite not being routinely recommended in clinical practice, when considering the patient's own wishes and the risks of the operation. Finally, As the majority of patients in this study were treated with PD-1 monotherapy, and fewer patients received other treatment regimens, our ability to assess the impact of different treatment protocols, particularly dual ICI combinations, on the development of myocarditis was limited. Further research is necessary to identify reliable indicators and optimize the combination of multiple indicators to enhance the accuracy of disease assessment.

## 5 Conclusions

Among the four simple and economical combination markers included in this study, AAR and LAR have the potential to identify severe myocarditis, and the SII, AAR, and LAR can be used to evaluate the prognosis of patients with ICI myocarditis. The increase in the SII, AAR and LAR during ICI treatment may indicate the risk of myocarditis. Surveillance of these biomarkers during ICI therapy, in addition to cardiac markers, might contribute to the risk prediction of ICI myocarditis as well as the assessment of disease severity and prognosis.

## Data Availability

The original contributions presented in the study are included in the article/Supplementary Material, further inquiries can be directed to the corresponding author.
