# Supplementary material for: Peripheral biomarkers to assess risk, severity, and prognosis of immune checkpoint inhibitor-associated myocarditis: a retrospective clinical study
Source: Front Cardiovasc Med. 2024 Oct 24;11:1465743. doi: 10.3389/fcvm.2024.1465743 (PMC11540693; doi:10.3389/fcvm.2024.1465743)
Supplement: Supplementary file 1 [file Datasheet1.docx]

| 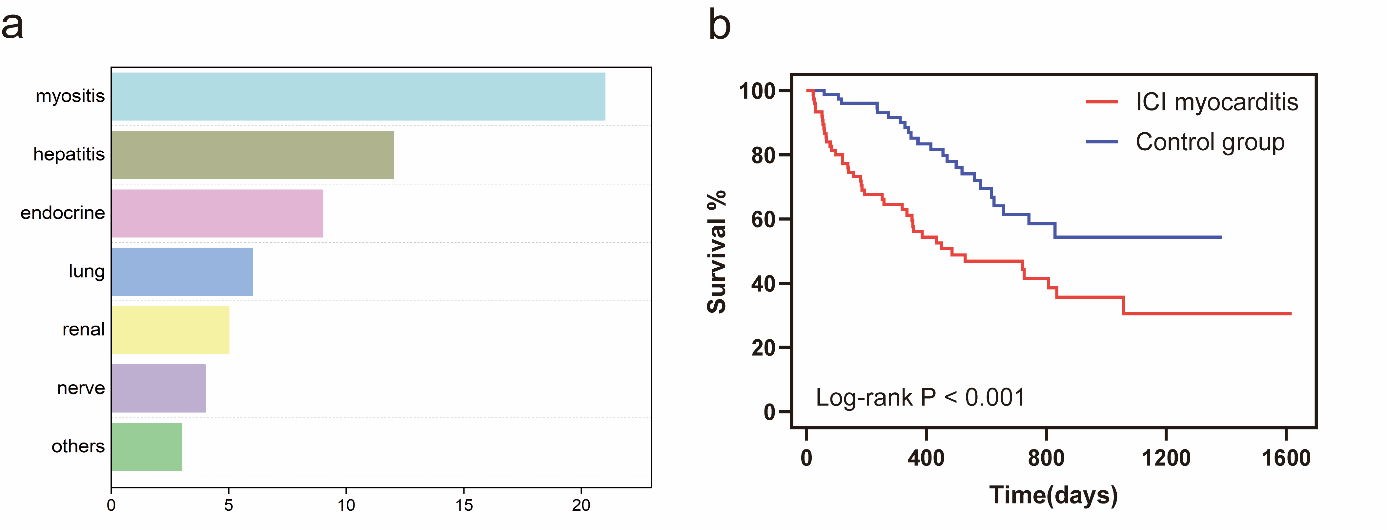 |
| --- |
| **Figure S1.** Patients with different types of other concomitant irAEs (A) and Kaplan–Meier analysis of all-cause mortality in patients with myocarditis and in control group (B). Abbreviations: ICI, immune checkpoint inhibitor; irAEs, immune-related adverse events. |

| 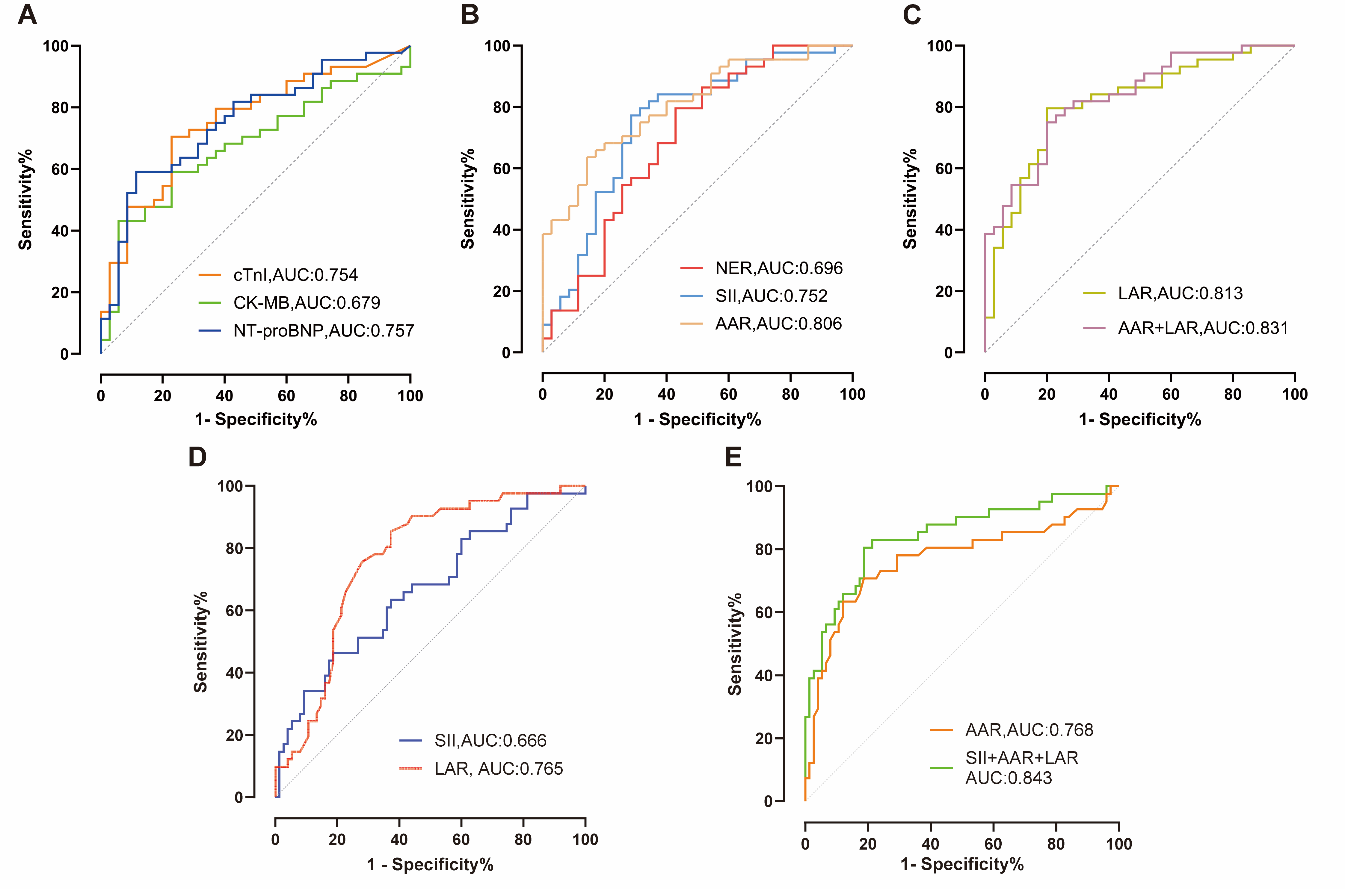 |
| --- |
| **Figure.S2.** ROC curves of biomarkers. A-C: The ROC curves of each biomarker and myocarditis severity are shown in three parts. A. ROC curves of cTnI, CK-MB, and NT-proBNP. B. ROC curves of SII, NER, and AAR. C. ROC curves of LAR and combine LAR+AAR. D. ROC curves of SII and LAR for the occurrence of myocarditis. E. ROC curves of AAR and combined application of SII, AAR, and LAR for the occurrence of myocarditis. Abbreviations: SII, systemic immune-inflammation index; NER, neutrophil to eosinophil ratio; AAR, aspartate transferase to albumin ratio; LAR, lactic dehydrogenase to albumin ratio; CK-MB, creatine kinase isoenzyme; cTnI, cardiac troponin-I; NT-proBNP, N-terminal pro-brain natriuretic peptide. |
