# Supplementary material for: Peripheral biomarkers to assess risk, severity, and prognosis of immune checkpoint inhibitor-associated myocarditis: a retrospective clinical study
Source: Front Cardiovasc Med. 2024 Oct 24;11:1465743. doi: 10.3389/fcvm.2024.1465743 (PMC11540693; doi:10.3389/fcvm.2024.1465743)
Supplement: Supplementary file 2 [file Datasheet2.docx]

Table S1. Cox regression analysis of Biomarkers related to the overall survival of ICI myocarditis in severe cases.

| Biomarkers at  myocarditis onset | unadjusted | | Adjusted^a^ | |
| --- | --- | --- | --- | --- |
|  | HR (95% CI) | *p*-value | HR (95% CI) | *p*-value |
| SII | 1.000(1.000,1.000) | 0.34 | 1.000(1.000,1.001) | 0.013 |
| NER | 1.001(1.000,1.002) | 0.021 | 1.001(1.000,1.002) | 0.032 |
| AAR | 1.044(1.020,1.069) | <0.001 | 1.050(1.021,1.079) | <0.001 |
| LAR | 1.025(1.010,1.041) | <0.001 | 1.021(1.005,1.037) | 0.011 |
| CK-MB, U/L | 1.000(0.997,1.001) | 0.257 | - |  |
| cTnI, ng/ml | 1.072(1.025,1.122) | 0.003 | 1.085(1.030,1.142) | 0.002 |
| NT-pro-  BNP, ng/L | 1.000(1.000,1.000) | 0.004 | 1.000(1.000,1.000) | 0.063 |
| ^a^Adjusted for age, BMI, other irAEs, and tumor stage.  Abbreviations: ICIs, immune checkpoint inhibitors; irAEs, immune-related adverse events; HR, hazard ratio; SII, systemic immune-inflammation index; NER, neutrophil to eosinophil ratio; AAR, aspartate transferase to albumin ratio; LAR, lactic dehydrogenase to albumin ratio; CK-MB, creatine kinase isoenzyme; cTnI, cardiac troponin-I; NT-proBNP, N-terminal pro-brain natriuretic peptide. | | | | |

| Table S2. Comparison of clinical characteristics between patients with myocarditis and controls | | | |
| --- | --- | --- | --- |
| Characteristics | ICI myocarditis  N=79 | Control group  N=158 | *p*-value |
| Age | 63.94±10.09 | 61.27±10.70 | 0.068 |
| Male | 58(73.4%) | 108(68.4%) | 0.422 |
| BMI, kg/m^2^ | 23.44(3.77) | 22.84±3.62 | 0.242 |
| Smoking history | 34(43.0%) | 62(39.2%) | 0.575 |
| Drinking history | 14(17.7%) | 30(19.0%) | 0.813 |
| CAD | 11(13.9%) | 16(10.1%) | 0.386 |
| Hypertension | 23(29.1%) | 63(39.9%) | 0.104 |
| Diabetes | 13(16.5%) | 27(17.1%) | 0.902 |
| Tumor type |  |  | 0.342 |
| Lung cancer | 23(29.1%) | 46(29.1%) |  |
| Esophageal carcinoma | 19(24.1%) | 32(20.3%) |  |
| Gastric cancer | 15(19.0%) | 46(29.1%) |  |
| Other tumors | 22(27.8%) | 34(21.5%) |  |
| Tumor stage |  |  | 0.164 |
| ≤III | 40(50.6%) | 95(60.1%) |  |
| IV | 39(49.4%) | 63(39.9%) |  |
| Therapy mode |  |  |  |
| Combined chemotherapy | 64(81.0) | 140(88.6%) | 0.111 |
| Combined targeted-therapy | 14(17.7%) | 38(24.1%) | 0.267 |
| Combined radiotherapy | 12(15.2%) | 22(14.0%) | 0.808 |
| Type of ICIs |  |  | 0.881 |
| Anti-PD-1 | 71(89.9%) | 141(89.2%) |  |
| Others | 8(10.1%) | 17(10.8%) |  |
| Abbreviations: ICIs, immune checkpoint inhibitors; BMI, body mass index; CHD, coronary heart disease, PD-1 = programmed cell death protein 1, PD-L1 = programmed death-ligand 1. | | | |

| Table S3. Comparison of clinical characteristics between patients with myocarditis and controls after propensity score matching | | | |
| --- | --- | --- | --- |
| Characteristics | ICI myocarditis  N=75 | Control group  N=75 | *p*-value |
| Age | 63.27±9.86 | 63.49±8.19 | 0.879 |
| Male | 54(72.0%) | 53(70.7%) | 0.857 |
| BMI, kg/m^2^ | 23.49±3.81 | 23.64±3.72 | 0.803 |
| Smoking history | 32(42.7%) | 32(42.7%) | 1.000 |
| Drinking history | 13(17.3%) | 15(20.0%) | 0.675 |
| CAD | 9(12.0%) | 8(10.7%) | 0.797 |
| Hypertension | 22(29.3%) | 27(36.0%) | 0.384 |
| Diabetes | 13(17.3%) | 14(18.7%) | 0.832 |
| Tumor type |  |  | 0.983 |
| Lung cancer | 23(30.7%) | 24(32.0%) |  |
| Esophageal carcinoma | 18(24.0%) | 16(21.3%) |  |
| Gastric cancer | 14(18.7%) | 14(18.7%) |  |
| Other tumors | 20(26.7%) | 21(28.0%) |  |
| Tumor stage |  |  | 0.414 |
| ≤III | 37(49.3%) | 42(56.0%) |  |
| IV | 38(50.7%) | 33(44.0%) |  |
| Therapy mode |  |  |  |
| Combined chemotherapy | 64(85.3) | 64(85.3%) | 1.000 |
| Combined targeted-therapy | 14(18.7%) | 13(17.3%) | 0.832 |
| Combined radiotherapy | 12(16.0%) | 7(9.3%) | 0.220 |
| Type of ICIs |  |  | 0.575 |
| Anti-PD-1 | 67(89.3%) | 69(92.0%) |  |
| Others | 8(10.7%) | 6(8.0%) |  |
| Medication cycles | 2(1,3) | 5(3,6) | <0.001 |
| All-cause mortality | 41(54.7%) | 23(30.7%) | 0.003 |
| Abbreviations: ICIs, immune checkpoint inhibitors; BMI, body mass index; CHD, coronary heart disease, PD-1 = programmed cell death protein 1, PD-L1 = programmed death-ligand 1. | | | |

| Table S4 Comparison of biomarkers between patients with ICI myocarditis and control group at baseline | | | |
| --- | --- | --- | --- |
| Biomarkers at baseline | ICI myocarditis | Control group | *p*-value |
| SII | 648.27(522.75,806.33) | 620.50 (442.57,894.23) | 0.865 |
| NER | 45.00 (20.32,105.96) | 37.69 (18.08,73.00) | 0.436 |
| AAR | 0.57(0.42,0.69) | 0.50 (0.40,0.59) | 0.181 |
| LAR | 4.58 (3.97,5.98) | 4.48 (3.86,5.22) | 0.314 |
| Abbreviations: SII, systemic immune-inflammation index; NER, neutrophil to eosinophil ratio; AAR, aspartate transferase to albumin ratio; LAR, lactic dehydrogenase to albumin ratio. | | | |

| Table S5. Comparison of biomarkers before the last ICIs and at baseline in patients with ICI myocarditis and control group | | | | |
| --- | --- | --- | --- | --- |
| Biomarkers before last ICIs | ICI myocarditis | *p*-value | Control group | *p*-value |
| SII | 727.14(510.99,1111.69) | 0.009 | 612.64(427.95,917.56) | 0.054 |
| NER | 53.67(18.89,166.19) | 0.019 | 44.87 (22.93,90.13) | 0.127 |
| AAR | 0.74(0.55,0.92) | <0.001 | 0.51 (0.43,0.73) | 0.377 |
| LAR | 5.35(4.69,7.63) | <0.001 | 4.36 (3.89,5.63) | 0.325 |
| Abbreviations: see Table S3. | | | | |
